# Tackling the long-tailed challenge of greenhouse tomato cultivation cycles recognition: a sub-group guided, multi-expert lightweight framework

**DOI:** 10.3389/fpls.2025.1571853

**Published:** 2025-09-05

**Authors:** Ruochen Zhang, Jingxin Yu, Lin Han, Huankang Cui, Lichun Wang, Fan Xu, Xiaoming Wei

**Affiliations:** ^1^ National Engineering Research Center for Intelligent Equipment in Agriculture, Beijing, China; ^2^ School of Horticulture and Landscape Architecture, Tianjin Agricultural University, Tianjin, China; ^3^ School of Mechanical and Automotive Engineering, Xiamen University of Technology, Fujian, China

**Keywords:** long-tail recognition, multi-expert grouping, lightweight model, mobilevit, greenhouse tomato cultivation cycles

## Abstract

**Introduction:**

Greenhouse tomato cultivation cycles recognition is often impeded by the long-tailed challenge, arising from significant differences in cycle lengths affecting data distribution. This imbalance hinders accurate recognition, particularly for rare stages, limiting intelligent management in precision agriculture.

**Methods:**

This study proposes a lightweight framework integrating a novel multi-expert grouping strategy with knowledge distillation. The dataset is divided into three groups (Head, Balanced, Tail) based on sample quantity. Separate expert models are trained on each group. Knowledge distillation then transfers the expertise of these models to a lightweight student model (MSC-MobileViT). MSC-MobileViT enhances the MobileViT foundation by incorporating a multi-scale convolution module to improve feature extraction across different scales, capturing both local details and global structure.

**Results:**

Experimental results demonstrate superior performance. The framework achieves an overall accuracy of 95.99%, precision of 91.03%, recall of 93.57%, and F1-score of 92.02%, outperforming state-of-the-art models (ResNet50, MobileNetV3, MobileViT variants). Crucially, it excels in handling tail classes, improving accuracy from 79.27% (baseline) to 93.83% for rare stages like "Substrate Soaking" and "Early Production". The maximum performance gap across categories is minimized to only 3.49 percentage points. The student model achieves this high performance while maintaining an extremely low parameter count (0.95M).

**Discussion:**

The proposed framework effectively addresses the long-tailed recognition challenge in greenhouse tomato cultivation cycles. The multi-expert grouping strategy optimizes learning for different data distributions, while knowledge distillation enables high performance within a lightweight model suitable for edge deployment. The integration of multi-scale convolution significantly enhances feature extraction in complex agricultural scenes. This research provides a new paradigm for long-tail recognition in agriculture and demonstrates the viability of deploying efficient, high-accuracy intelligent systems in real-world greenhouse environments.

## 1 Introduction

As the global population grows and climate change intensifies, ensuring food security has become a significant challenge for contemporary societies. In this context, intelligent greenhouse production, as a model of modern precision agriculture, is revolutionizing the traditional agricultural production model. Among them, tomato, as one of the most economically valuable vegetables in the world (Bergougnoux, 2014), is rich in vitamins C and E, as well as a variety of natural pigments and flavonoids that help to prevent oxidative damage and other minerals and antioxidants that are beneficial to the human body (Dorais et al., 2008), which makes it a high-quality food to support healthy lifestyles and an innovative agricultural technology development. Along with these technological advances, tomato cultivation has transformed from traditional open-air cultivation to greenhouse cultivation, where precise control of environmental factors - such as temperature, humidity, light intensity, and carbon dioxide concentration - has significantly optimized the tomato cultivation cycles. Controlling environmental factors in the greenhouse - such as temperature, humidity, light intensity, and carbon dioxide concentration - it is possible to significantly optimize the cultivation cycles of tomatoes, thus ensuring continuity and sustainability of supply. This shift to a more controlled growing process has led to an increase in tomato yields and quality.

**Figure 1:**
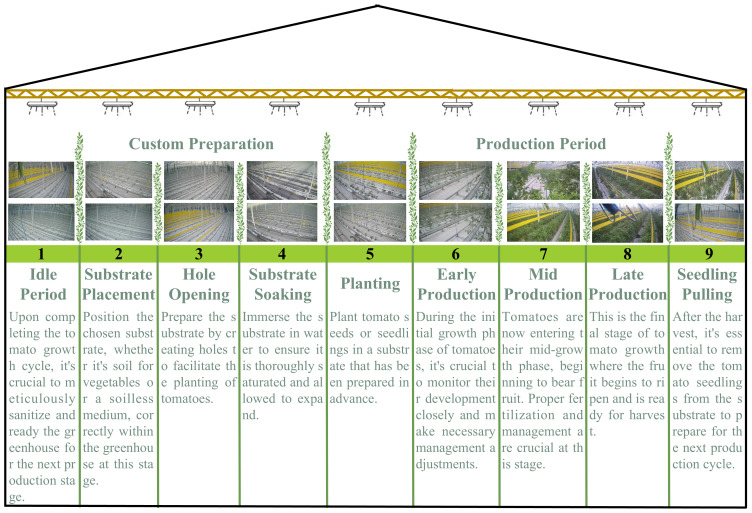
Greenhouse tomato cultivation cycles.

Accurate identification of cultivation cycles at each stage of greenhouse tomato growth is the basis for realizing intelligent management, and its production process covers nine key steps, as shown in **Figure 1**. Greenhouse tomato, such as substrate placement, hole opening, soaking, etc. The precise execution of these steps is crucial for ensuring the quality of production. In the whole production system, the accurate identification and monitoring of growing stages is vital, which involves the real-time monitoring of the internal environment of the greenhouse and the tracking of tomato cultivation cycles and decision support. By accurately monitoring cultivation cycles, producers can adjust management strategies, such as irrigation or fertilization programs, promptly to adapt to changes in the environment and the specific growth needs of the crop. This dynamic management improves production efficiency, enhances adaptability to environmental changes, and ensures efficient and sustainable production. In particular, data from greenhouse tomato production shows a significant long-tailed distribution, with extreme variations in the duration of different cultivation cycles. This poses a challenge to traditional machine learning and computer vision methods. For example, “mid-production” may last for months while “substrate soaking” takes only a few hours, and this unbalanced data distribution may affect the model’s overall performance and lead to misclassification of a few classes. This extremely unbalanced data distribution significantly challenges traditional machine learning and computer vision methods. The long-tailed distribution problem not only affects the model’s overall performance but also may lead to serious misclassification of a few classes, thus affecting the accuracy of production decisions.

Various solutions have been proposed in the academic community to address the long-tailed distribution problem. From the data level, methods such as oversampling (Dablain et al., 2022), undersampling (Hasegawa and Kondo, 2022), and data augmentation (Yun et al., 2019; Zhang, 2017) attempt to balance the data distribution directly. Algorithmic-level improvements include cost-sensitive learning (Thai-Nghe et al., 2010), focus loss (Ross and Dollár, 2017), and class balancing loss (Cui et al., 2019). Integration learning methods such as Cascade-RCNN (Cai and Vasconcelos, 2019) and PISA (Weng and Luo, 2023) improve performance by combining multiple models. Migration learning (Liu et al., 2022; Zhou et al., 2020) and meta-learning (Guo et al., 2022; Shu et al., 2019), on the other hand, try to address the category imbalance problem from different angles. However, these methods still face many challenges in practical applications, such as high computational complexity, limited generalization ability, and susceptibility to overfitting.

In recent years, Mixture of Experts (MoE) has shown great potential in dealing with complex data distributions, and the core idea of MoE is to decompose a complex task into multiple sub-tasks, each handled by a specialized “expert” model. This approach was first proposed by Jacobs et al. (1991) and has been widely used in deep learning. For example, Sparsely-Gated MoE proposed by Shazeer et al. (2017) significantly improves the performance of large-scale language models, and AdaMV-MoE by Chen et al. (2023) makes breakthroughs in the ImageNet classification task. The MoE approach has a unique advantage in dealing with long-tailed data: it can train specialized expert models for different data distribution features. Different data distribution features are used to train specialized expert models, adaptively select the most suitable experts through a dynamic routing mechanism, and have good scalability and interpretability. Therefore, the MoE method shows higher potential and flexibility than traditional methods in solving the long-tailed distribution problem.

However, the direct application of MoE to greenhouse tomato cultivation cycles identification still faces many challenges. Firstly, the traditional MoE method requires significant computational resources, which is unfavorable to be deployed in resource-constrained natural production environments. Secondly, how to effectively coordinate the knowledge interaction between different expert models to avoid the phenomenon of “expert monopoly” is still an open problem. Finally, it is also a challenge to realize the lightweight model while maintaining high performance to adapt to the needs of edge computing devices.

Based on the above analysis, this study proposes an innovative multi-expert grouping enforcing strategy coupled with a lightweight model, aiming to solve the problem of long-tail identification of greenhouse tomato cultivation cycles. Our approach includes the following key innovations:

1. A novel data grouping strategy is proposed to divide the long-tailed distribution dataset into three groups, head, balance, and tail, according to the number of samples and train the expert models separately. This strategy optimizes the model for different distribution features and effectively alleviates the category imbalance problem.
2. Knowledge distillation technique is introduced to effectively transfer the knowledge of multiple expert models into one lightweight student model. This step reduces the model complexity, retains the advantages of the expert models, and realizes the balance between high performance and low number of parameters.
3. Introducing a multi-scale convolution module on top of MSC-MobileViT significantly enhances the feature extraction capability of the model. This improvement enables the model to focus on both local details and the image’s global structure, improving the accuracy of cultivation cycles recognition in complex scenes.

To validate the effectiveness of the proposed method, we constructed a large-scale greenhouse tomato image dataset containing nine cultivation cycle categories, covering the entire cultivation cycle from planting preparation to ripening and harvesting. We conducted comparative experiments of the proposed method with various state-of-the-art baseline models, including traditional CNN models (e.g., ResNet50, MobileNetV3) and models designed for lightweight applications (e.g., MobileViT series). The experimental results comprehensively evaluate the model performance in terms of several metrics, such as accuracy, precision, recall, and F1 score, mainly focused on the model’s performance in processing tail categories. In addition, we conducted in-depth ablation experiments to analyze the respective contributions of the multi-expert strategy, knowledge distillation, and multi-scale convolution modules. Through visual analysis, we further explore the decision-making mechanism of the model, providing new insights for understanding and improving the processing of long-tailed distribution data.

The significance of this study is not only limited to improving the intelligence of greenhouse tomato production but also provides new ideas for solving the long-tailed identification problem prevalent in agriculture and industry. We expect that this work will promote the development of intelligent agriculture technology and contribute to realizing more efficient and sustainable agricultural production. In the following sections, we will introduce the proposed method, experimental design, result analysis, and the outlook of future research direction in detail; the contents of this paper are organized as follows: Section 2 describes the related work and research background of this study; Section 3 describes the details of our dataset; Section 4 describes the overall structure and details of this method; Section 5 reports the experimental results and evaluation; Section 6 describes the conclusions of this paper and prospects for future work.

## 2 Related work

In the current tomato factory production, cultivation cycles identification faces several challenges: 1) Due to the significant variation in the lengths of various cultivation periods, there is a long-tailed distribution of the collected data, which poses a significant challenge for data analysis and model training. Long-tailed distribution implies that a large amount of data is concentrated in a few categories. In contrast, most categories have only a small amount of data, making it difficult to thoroughly learn the features of all the categories when the model is trained, which affects the model’s generalization ability. Existing feature extraction methods also show limitations when dealing with long-tailed distribution data, as they are often based on the assumption that the data is uniformly distributed, which is inconsistent with many reality conditions. 2) Significance area feature extraction is also a challenge that needs to be solved, especially in the case of high scene similarity, where identifying the exact region becomes more difficult. This requires models that accurately extract and recognize target features from similar backgrounds. 3) To adapt to the practical demands of greenhouse production, lightweight models must be developed to ensure deploy ability. These models need to reduce the consumption of computational resources while maintaining high accuracy to run efficiently on various hardware platforms for real-time monitoring and analysis. The solution to these challenges will promote innovative agriculture development and improve the efficiency and quality of tomato factory production. Therefore, this study investigates the existing problems:

### 2.1 Long-tail identification

Long-tail identification, as a critical challenge in the current greenhouse cultivation cycles recognition, has caused significant difficulties in data collection due to the large difference in the duration period of different stages, in which case the category imbalance problem is particularly prominent, as the data of a few categories are often challenging to obtain, which directly affects the training and performance of the model. This class of problems has now been provided with category rebalancing (Hong et al., 2021; Park et al., 2021; Wu et al., 2021; Zhang and Pfister, 2021), information enhancement (He et al., 2021; Kim et al., 2020; Yin et al., 2019) and network structure improvement (Kang et al., 2020; Wu et al., 2020; Zhong et al., 2021; Zhu and Yang, 2020) are the three paradigms on which the researchers designed a series of improvement strategies for the long-tailed recognition problems faced in different agricultural scenarios.

Zhang et al. (2023b) solved the long-tail recognition problem of food crop disease images based on migration learning with a Bilateral-Branch Network (BBN) as a framework. They contributed three re-sampling strategies, finally achieving 94.3% recognition accuracy on the long-tail dataset of food crop disease images. Sun et al. (2021) argued that the decoupled representation and classifier algorithm is the crucial method to solve the long-tailed recognition problem and proposed a two-channel algorithm based on decoupled representation and classifier, which utilizes two channels to focus on the head class and the middle-tail class respectively. The algorithm achieves an accuracy, precision, and recall of 93.81%, 94.27%, and 90.80%, respectively, for the peach leaf disease recognition task. The recognition accuracy of the head, middle, and tail classes is 93.81%, 94.27%, and 90.80%, respectively. Head, middle, and tail classes were 94.21%, 90.13%, and 88.57%, respectively. Saleh et al. (2023) proposed a new method for weed comparison learning through visual representations, WeedCLR, which utilizes class-optimized loss and the von Neumann entropy of the deep representations. Neumann Entropy) for classifying weeds in long-tailed datasets. All of the above methods provide valuable references, but they cannot meet the lightweight requirement of a tomato cultivation cycles recognition system; in addition to that, this study needs to consider the problem of highly similar work environments, in other words, in mining the recognizable features of different cultivation cycles at the exact location.

### 2.2 Significance area feature extraction

Saliency feature extraction is a crucial step in studying greenhouse tomato g cultivation cycles recognition. It is challenging as the above process differentiates between different working cultivation cycles in the same scene. This work was done in the early days mainly by extracting features manually (Cheng et al., 2014; Oliva and Torralba, 2006), which relied heavily on people’s prior knowledge and was time-consuming and labor-intensive. With the rapid rise of deep learning, saliency region feature extraction methods have achieved significant breakthroughs in computer vision, with major advances including attention mechanisms, multi-scale, and feature fusion.

Attention mechanisms aim to focus attention on essential features in an image (Hermann et al., 2015), mainly including channel attention (Hu et al., 2018; Wang et al., 2020), spatial attention (Hsieh et al., 2019; Hu et al., 2020; Woo et al., 2018) and self-attention (Parmar et al., 2018; Vaswani et al., 2017), which are also widely used in agriculture. Facing the problem of small spot size in citrus disease identification, which makes it difficult to focus and extract feature information, Zhang et al. (2024) proposed a frequency-domain attention network (FdaNet), which changes the weight of each frequency domain by adaptively learning the importance of the feature information between different frequency domains during the network inference process. Zhang et al. (2023a) added a YOLO feature pyramid structure by adding the attention mechanism module (ECA-Net) and adaptive feature fusion mechanism (ASFF), which effectively solves the problems of small size of budgerigar, limited features, and unclear attributes. Sun et al. (2023) chose the M2-transformer network as the decision base generator. They proposed a method named “DFYOLOv5m- M^2^Transformer”, a two-stage image-dense annotation model, which can generate visual disease feature description sentences based on identifying the disease region.

The attention mechanism is essential in improving model performance, especially when dealing with complex tasks. However, this mechanism increases the computational burden (Hassanin et al., 2024) and may affect the speed and flexibility of model deployment. In contrast, multiscale feature fusion techniques, which can integrate information at different levels and provide a more comprehensive view of the data while controlling the computational complexity, which is particularly important for real-time applications and resource-constrained environments, were first proposed by Szegedy et al. (2015). Since then, multiscale feature fusion techniques have been widely used in many fields. Zhao et al. (2022) constructed a multiscale feature fusion network consisting of ResNet, FPN, and CBAM blocks, which can effectively extract rich disease features in strawberry leaves, flowers, and fruits. Rong et al. (2020) proposed a segmentation method based on a multiscale residual fully convolutional network in the pecan impurity detection task to overcome the complexity of the foreign object’s shape and color in different postures. Challenges. Subsequently, to meet the demand for lightweight deployment of the model, this study also investigates a lightweight visual transformer.

### 2.3 Progress in lightweight transformer structure research

Visual Transformer’s (ViT) success is attributed to the multi-head attention module. At the same time, its significant model parameters and high latency make it unsuitable for deployment on resource-constrained devices. As a result, researchers have successively proposed a series of lightweight backbones for ViTs. MobileViT (Mehta and Rastegari, 2021) is one of the typical success stories, which implicitly integrates global representations by using the transformer as a convolution, combining the strengths of the CNN over the ViT, i.e., the multi-head self-attention and spatial inductive bias, and allowing them to learn representations with only a small number of parameters. Graham et al. (2021) downplayed the notion of a token in the transformer in their proposed LeViT while introducing the activation map in CNNs and designing computationally efficient image chunk extractors that can reduce the number of features in the first layer. Chen et al. (2022) proposed a MobileNet and Transformer parallelization of Mobile-Former, stacking mobile blocks with images as inputs and using efficient depthwise and pointwise convolution to extract pixel-level local features in ImageNet classification task from 25–500 MFLOPs under the stringent regime of MobileNetV3 (Howard et al., 2019). Vasu et al. (2023) argued that the research on efficient networks should not focus only on minimizing the FLOPs or the number of parameters, as there is no strict consistency between these two and the inference efficiency. An efficient and generalized backbone network for mobile devices, Mobileone, is proposed, which uses a model extension strategy with a parameterizable structure to obtain advanced performance, achieving 75.9% Top1 accuracy on the ImageNet dataset with a speed of< 1 ms. Liu et al. (2023) found that memory access overhead is a key factor affecting the model’s speed; the proposed EfficientViT uses a single memory-bound MHSA between efficient FFN layers, improving memory efficiency while enhancing channel communication. The above study opens up a new scope for lightweight applications of ViTs.

Wang et al. (2024) then proposed a forest fire segmentation model, FireViTNet, based on MobileViT, which not only achieved an F1 score of 87.2% but also ensured the model’s lightweight and deployability. In a study of citrus green fruit detection for real-world applications, Lu et al. (2023) used the strategy of YOLOv5 combined with MobileViT to achieve an accuracy of 93.6% with only 6.3 M model parameters. mobileOne-YOLO (Li et al., 2023) is a new method to detect unfertilized duck eggs and early duck embryo development, i.e., it is a combination of YOLO and YOLO to detect unfertilized duck eggs and early duck embryo development. A new method, i.e., replacing the backbone network of YOLOv7 with MobileOne, improved the FPS performance by 41.6 without loss of accuracy. This paper investigates a lightweight long-tail identification of greenhouse tomato work based on MobileViT.

## 3 Materials

### 3.1 Source of data set

In this study, data collection and processing are crucial parts that directly affect the accuracy and reliability of the research results. The data were obtained from Beijing Cuihu Workshop and Ulanqab Hongfu Modern Agricultural Industrial Park, which adopt advanced factory elevated soilless culture production mode and are equipped with intelligent greenhouse management systems. This model not only improves the efficiency of tomato production but also provides us with an ideal environment for data collection.

The data collection period is from May 2023 to April 2024, covering the entire tomato factory production cycle. The data from this period allows us to observe the changes in the cultivation cycles of the greenhouse during different seasons and cultivation cycles, which is essential for understanding the environmental demands of tomato growth and adjusting production strategies. The data collection involved nine critical stages from the idle period to seedling pulling, each with unique ecological parameters and production requirements, which are highly valuable for analyzing the dynamic changes of the environment inside the greenhouse and optimizing production management.

### 3.2 Data collection methods

The image acquisition system utilized 16 cameras (1920×1080 resolution) with three deployment configurations: fixed cameras for 24-hour continuous monitoring, mechanically adjustable cameras for multi-angle canopy imaging, and handheld devices for supplementary capture of hidden areas. All cameras were installed at varying heights to ensure full spatial coverage of the cultivation area.

Tomato cultivation cycles annotation combined automated and manual processes. Initial labels were generated through timestamp synchronization with greenhouse management system logs, followed by agronomic expert verification and supplementation for complex cases. A dual-operator cross-checking procedure was implemented to ensure labeling consistency.

### 3.3 Data pre-processing methodology

This study employed standardized data partitioning and augmentation protocols to ensure experimental reproducibility. The original dataset containing 1,999 images was divided into two mutually exclusive subsets through random stratified sampling at a 1:1 ratio, resulting in an initial training set of 1,002 images and a test set of 997 images. To ensure adequate model training, we implemented random image augmentation for categories containing fewer than 100 samples in the training set. The augmentation techniques included: 90° rotation, 180° rotation, contrast reduction, contrast enhancement, horizontal mirror flipping, Gaussian blurring, and Gaussian noise addition. Following these procedures, the final dataset consisted of 1,362 training images and 997 test images. All images were resized to 224 × 224 pixels before being input into the model. **Table 1** shows more details about the number of images in the dataset.

**Table 1:** Details of the dataset.

Duration	Number of raw images	Number of images in train set	Number of pictures in train set by augmentation	Number of images in the test set	Total number of images
Idle Period	608	304	304	304	608
Substrate Placement	59	30	90	29	119
Hole Opening	51	26	104	25	129
Substrate Soaking	32	16	96	16	112
Planting	113	57	114	56	170
Early Production	24	12	96	12	108
Mid Production	589	295	295	294	589
Late Production	263	132	132	131	263
Seedling Pulling	260	130	130	130	260

### 3.4 Analysis of the data distribution

**Figure 2:**
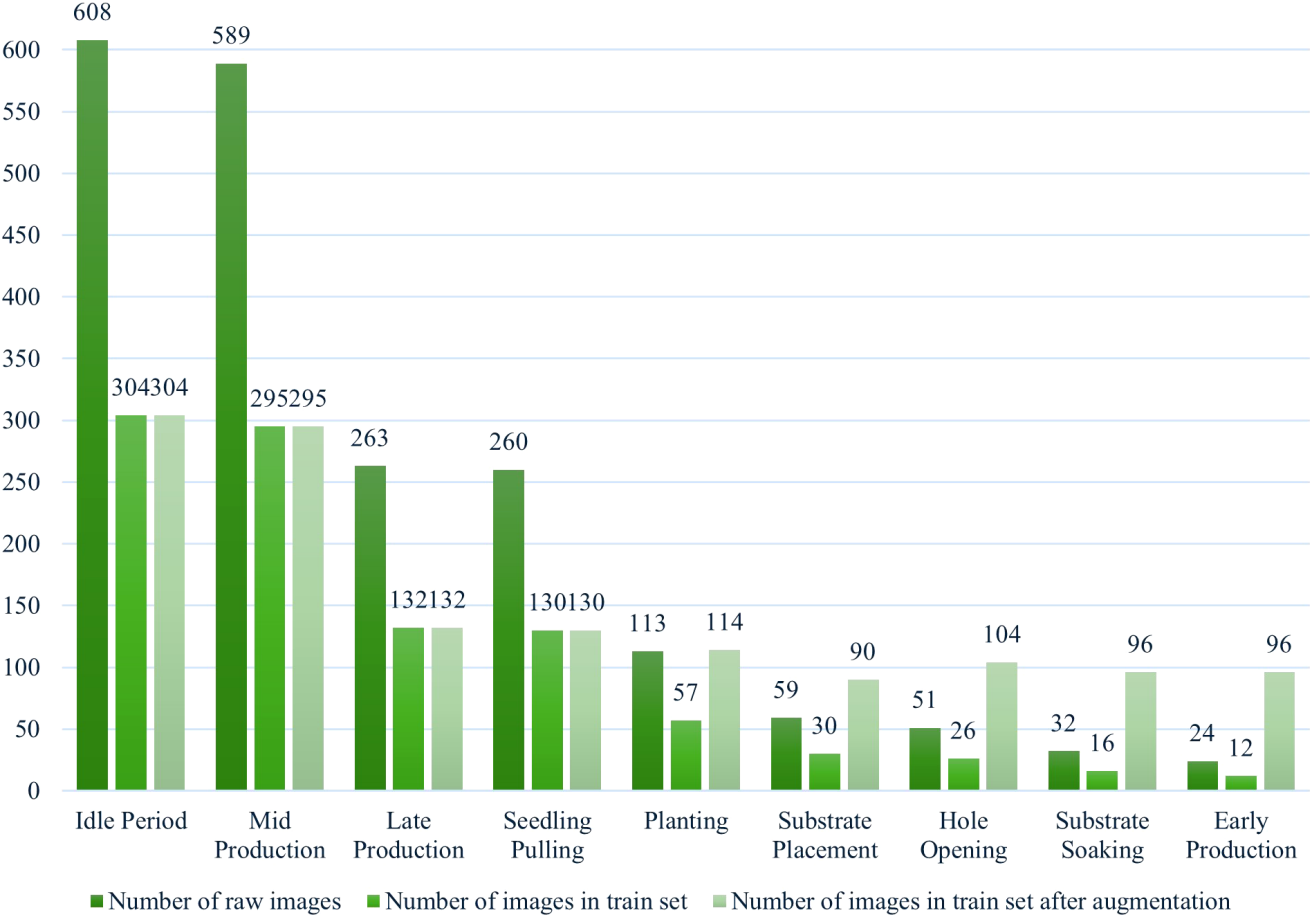
Data distribution.

Analyzing **Figure 2**, it is easy to see that even after the tail-category enhancement process, the dataset still has a long-tailed distribution because only a few categories in the dataset have many samples. In contrast, most other categories have a relatively small number of samples. This may cause the model to overfit on high-frequency categories and underfit on low-frequency categories during training. Overfitting means that the model may not be able to generalize to new, unseen data, while underfitting may result in poor model performance on specific categories. However, greenhouse tomato cultivation cycles are inherently highly variable, and it is difficult to obtain an idealized data distribution; therefore, long-tail identification is the focus of this study.

### 3.5 Analysis of data characteristics

**Figure 3:**
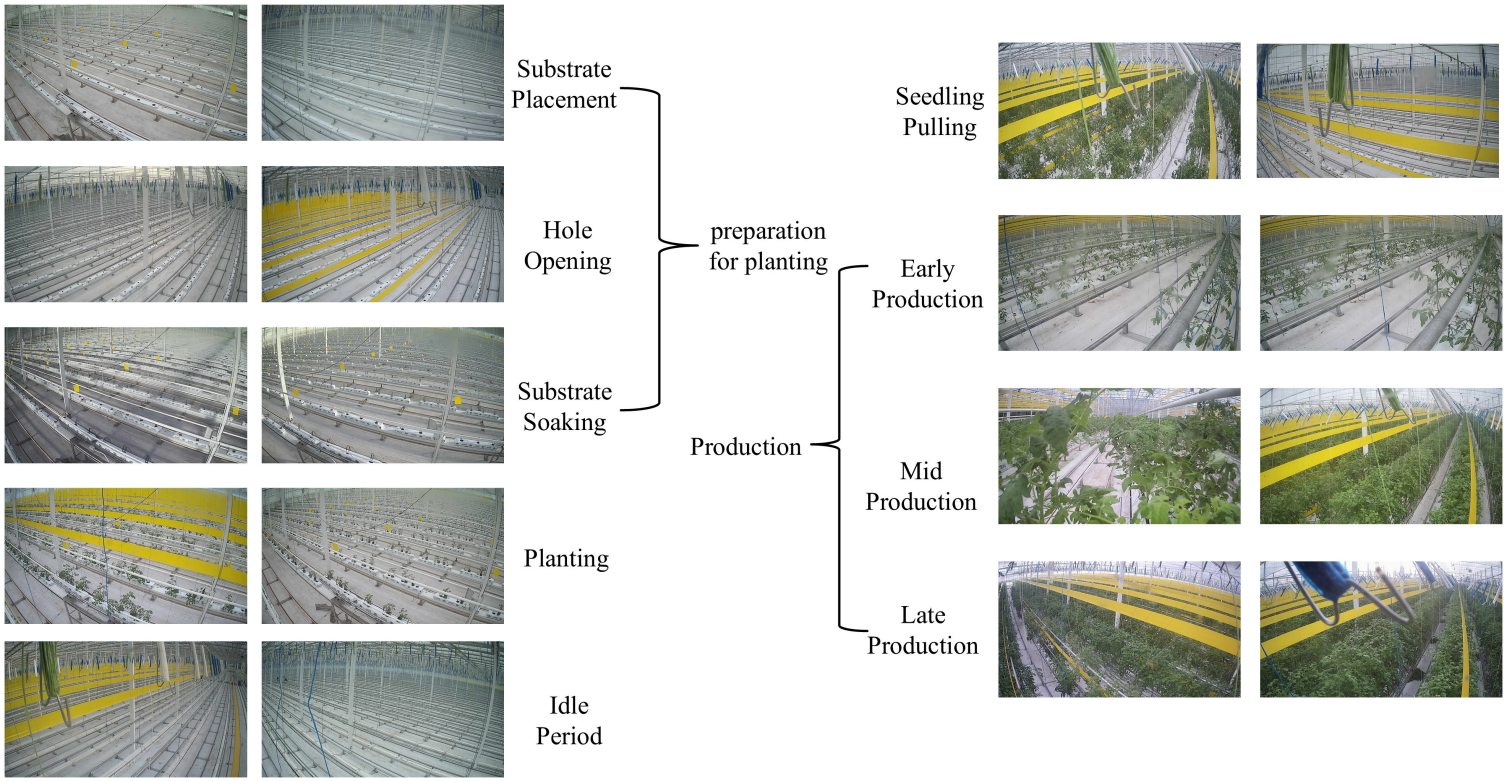
Part of the sample display.

**Figure 3** shows a brief view of the different categories of samples. Since all the samples are from the same greenhouse, there may be a lot of duplicated information between the different categories, especially in the neighboring phases. For example, in the “Preparation for planting” phase between “Substrate placement,” “Hole opening,” and “Substrate soaking,” only a few features can be used to distinguish between them, which makes the task as tricky as fine-grained image classification. The discriminative features appear at different scales in many samples, which places high demands on the robustness of the model and its ability to capture contextual information. In addition, there is a risk of information loss in both deep neural networks’ pre-processing and down-sampling stages. Therefore, feature extraction of saliency regions is also essential in this study.

## 4 The proposed methodology

**Figure 4:**
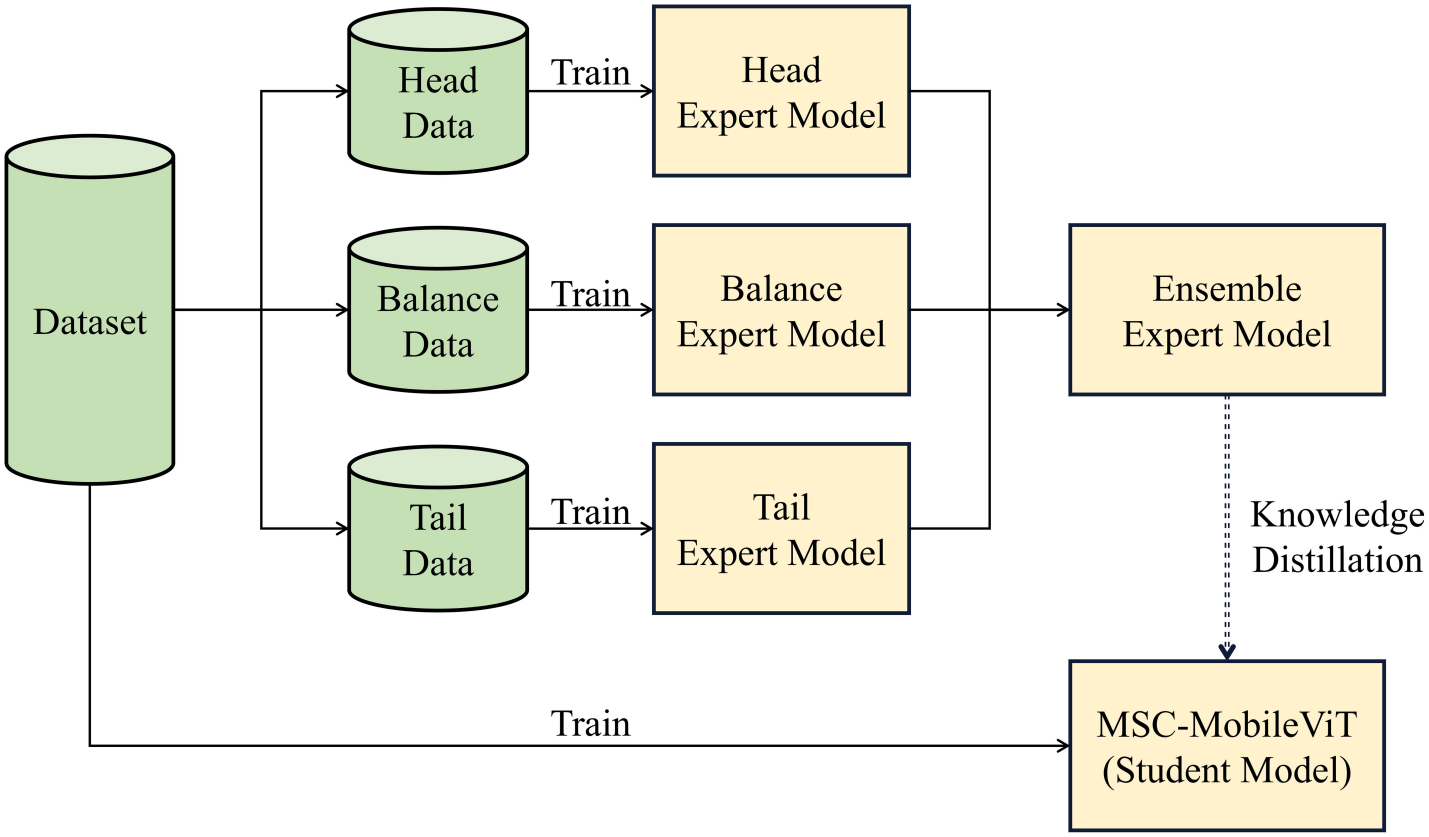
Process of the proposed methodology.

The dataset of the cultivation cycles recognition-related task inevitably shows a long-tailed distribution due to the significant difference in the length of the work period, which makes the model allocate more confidence to the head category to obtain higher accuracy, but due to the low accuracy of the tail category, it is prone to the situation that the indexes are too high but not able to satisfy the actual requirement. Therefore, this paper proposes a multi-expert joint group-guided long-tail recognition scheme, in which the categories in the dataset are firstly divided into three groups according to the number of samples, namely “head,” “balance” and “tail,” and each group is trained separately. Meanwhile, to optimize the salient feature extraction capability of the method and meet the lightweight deployment requirements, this paper proposes a multi-scale lightweight ViT model named MSC-MobileViT and lets the integrated expert model guide its training through knowledge distillation; **Figure 4** shows the specific flow of the method.

### 4.1 The multi-expert joint guidance methodology

In researching cultivation cycles recognition, we adopt an innovative joint approach of multi-expert models to effectively deal with the category imbalance problem existing in the training data. Specifically, we first analyzed the entire tomato cultivation cycles recognition dataset in detail, as shown in **Table 2**, and arranged the samples in descending order according to the number of samples in each category in the training set. Then, we divided the dataset into three different groups. This grouping strategy was initially designed to allow each expert model to focus on training a specific subset of data, thus avoiding model bias due to excessive samples in particular categories. In this process, we are particularly mindful that training only specific groups of categories can impair the generalization ability of the expert model. Therefore, we introduce the concept of open-set identification, a method that considers unknown categories during the model training phase. We categorize all samples that do not belong to the current group as “other” and include them in the model training. This aims to allow the model to learn the ability to distinguish between known and unknown categories so that when faced with subsequent integration and distillation tasks, it can more robustly categorize samples from other groups and more effectively guide the student model.

**Table 2:** Grouping data sets by sample size.

Stages	Number of images in train set	Number of pictures in train set after augmentation	Groups
Idle Period	304	304	Head
Mid Production	295	295	
Late Production	132	132	Balance
Seedling Pulling	130	130	
Planting	57	114	
Substrate Placement	30	90	Tail
Hole Opening	26	104	
Substrate Soaking	16	96	
Early Production	12	96	

**Figure 5:**
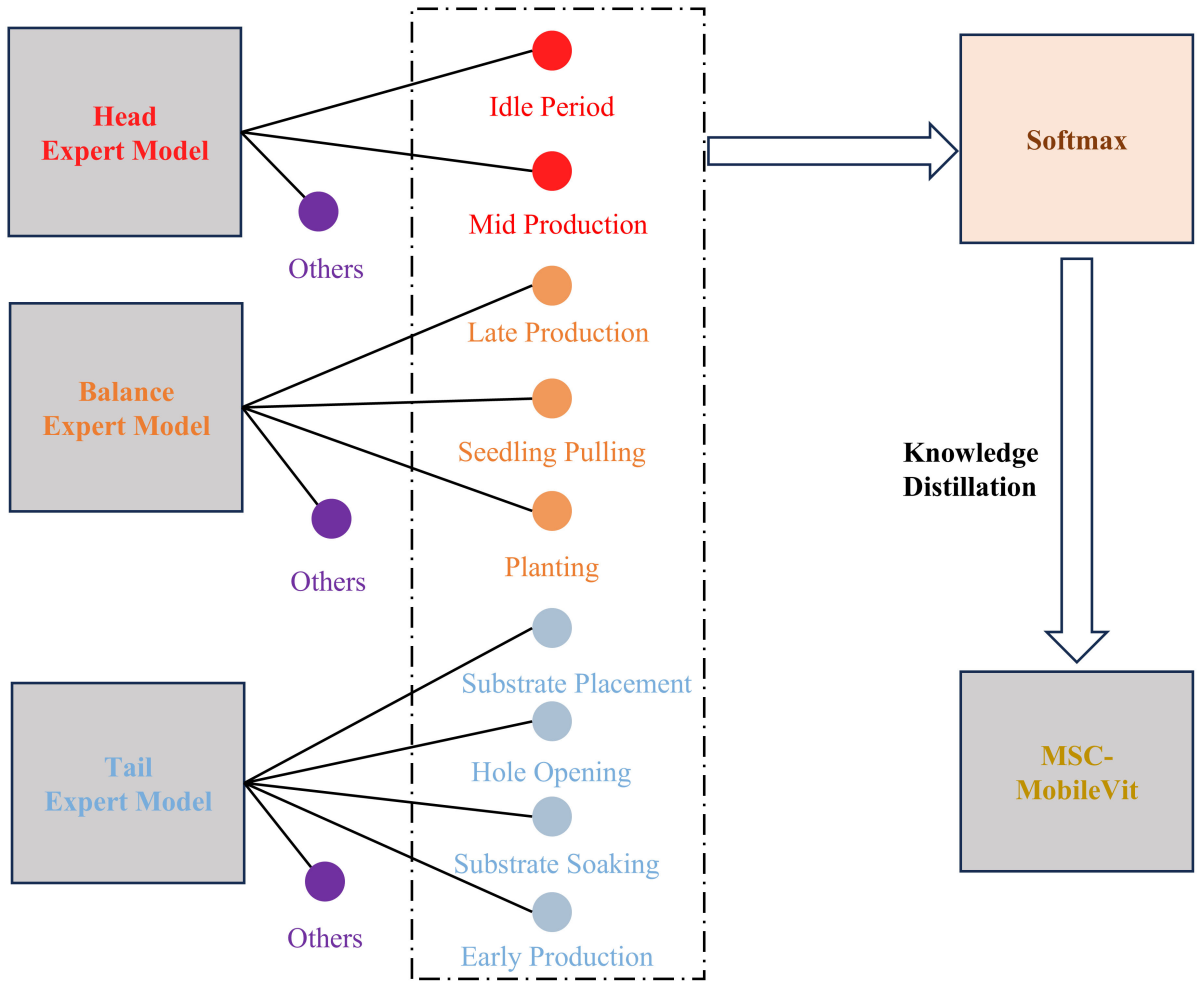
Sub-group guidance mechanism.

When constructing the expert models, to ensure adequate training even with a limited number of samples in the target classes, MobileViT-s are used as feature extractors for each specific category, which can increase the model’s sensitivity to a small number of features. In the model’s output layer, a distinctive node configuration is implemented, augmenting the number of target categories by one. This additional node serves as an “other” category, specifically designed to accommodate instances that do not fall within the predefined categories. As shown in **Figure 5**, this design allows for greater flexibility and robustness in the model’s classification. Once the three expert models have been trained, the next step is to integrate them. All the nodes except the “other” node are integrated in their category order in the above process. The outputs of these nodes are then processed by a softmax function, which generates a probability distribution representing the predictive confidence of each category. Ultimately, using the technique of knowledge distillation, these probability distributions are used as soft labels to guide the training of student models. In this way, the student model can learn rich feature knowledge from the expert model and reduce the impact of long-tailed distributions on its training. Section 4.2 will introduce the design idea of the student model MSC-MobileViT.

### 4.2 MSC-MobileViT

The design concept of the MSC-MobileViT model is to achieve a perfect balance between high accuracy and a low number of parameters to meet the needs of practical deployment. Although the model can improve its performance by guided training with multiple expert models, it must also enhance its capability in saliency region feature extraction. MobileViT-xxs, as its backbone network, introduces a self-attention mechanism that effectively handles contextual information while keeping the number of parameters inexpensive, facilitating its deployment in resource-constrained environments. In addition, the structural similarity between MobileViT-xxs and the three expert models provides an ideal basis for logit distillation, further enhancing the models’ performance. The innovation of this study on this basis is that replacing the first convolution module of the model with a multi-scale convolution not only enhances the model’s ability to capture features at different scales but also broadens the model’s potential to handle more complex visual tasks. The above improvements open up new possibilities for the model’s versatility and adaptability, enabling it to demonstrate enhanced performance and flexibility in the face of variable visual challenges.

**Figure 6:**
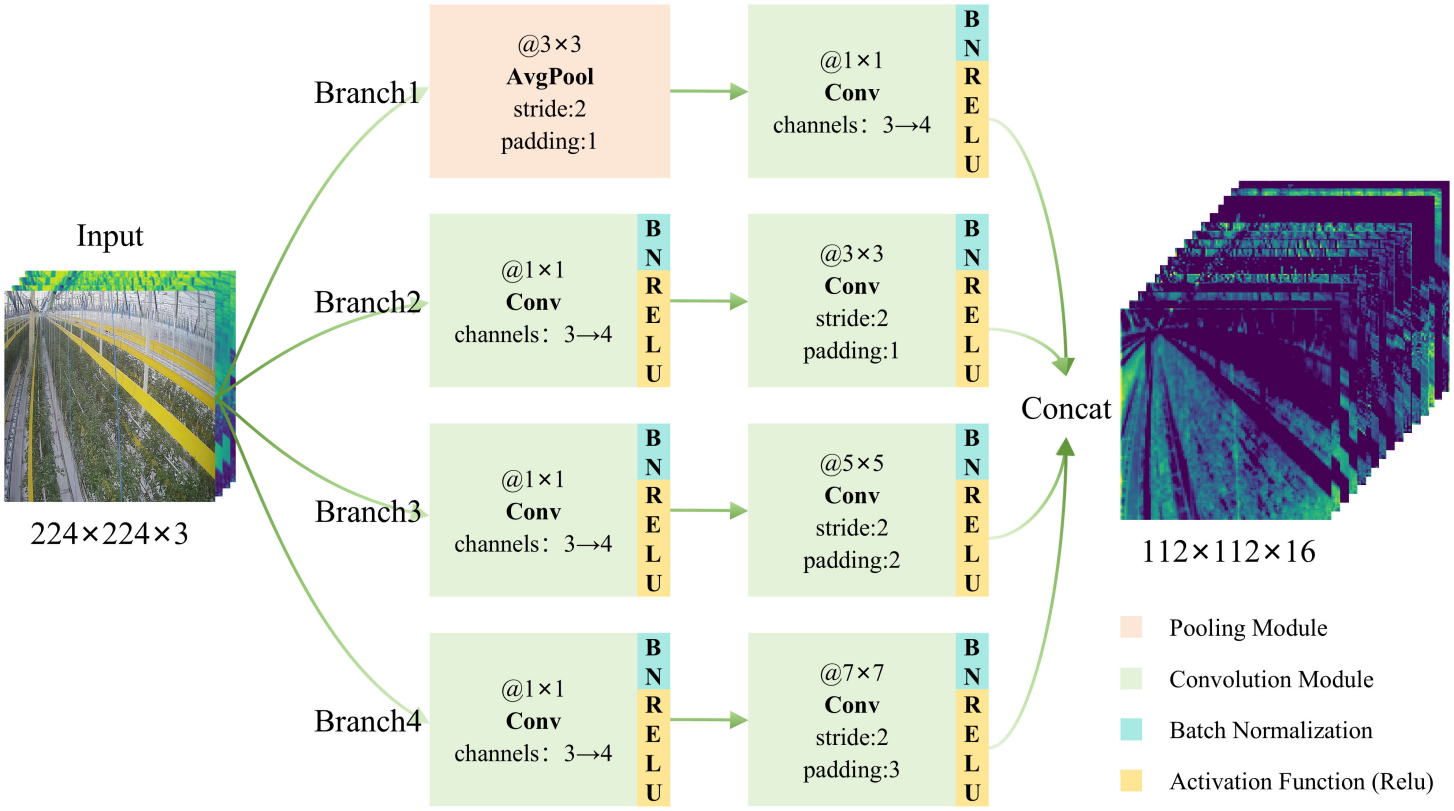
MultiScaleConv module.

The proposed MultiScaleConv module shown in **Figure 6** is designed to extract multi-scale features from the input image. The module consists of four independent branches, each performing convolutional operations at different scales to capture information from different-sized receptive fields. The first branch reduces the resolution of the feature map through a 3x3 average pooling layer, followed by feature upscaling using a 1x1 convolutional layer and enhancing the nonlinear properties through batch normalization and Relu activation functions. The second branch is initially designed to enhance the feature dimension by a 1x1 convolutional layer, followed by batch normalization and Relu activation application, and a 3x3 convolutional layer for further feature extraction. The third branch builds on this using a 5x5 convolutional kernel to capture a wider range of spatial features and apply batch normalization and Relu activation. Conversely, the fourth branch utilizes a 7x7 convolutional kernel to cover a more extensive range of receptive fields. The outputs of these branches are eventually merged in the channel dimension to form a composite feature map that fuses multi-scale features, greatly enhancing the model’s ability to fully understand and characterize the input image. This multiscale feature fusion strategy significantly improves the model’s adaptability to image scale changes and saliency region feature extraction capability.

### 4.3 Loss functions

The idea behind the group coaching mechanism is to transfer the expertise of the three expert models to MSC-MobileViT through knowledge distillation, which can be likened to matching a student with a specialized teacher in each subject area. First, each teacher model is spliced with the output nodes except the “other” node, softmax to obtain soft labels, and the temperature T is introduced to perform label smoothing in **Equation 1**:


(1) $$q_{i}=\frac{\text{exp}\left(z_{i}/T\right)}{\sum_{j}\text{exp}\left(z_{j}/T\right)}\;$$

Then, in **Equation 2**, the output of MSC-MobileViT is done the same way:


(2) $$p_{i}=\frac{\text{exp}\left(v_{i}/T\right)}{\sum_{j}\text{exp}\left(v_{j}/T\right)}$$

In **Equation 3**, the difference between the two feature distributions is compared by KL scatter to generate a soft loss:


(3) $$L_{soft}=-\sum_{i}^{n}p_{i}\log\left(q_{i}\right)$$

To avoid the expert model passing its error to MSC-MobileViT, then a hard label is obtained by comparing the probability distribution of the student with the real label through ReduceFocalLoss loss, and the parameter α is set to weight the sum of the two losses, as detailed in **Equations 4** and **5**:


(4) $$L_{hard}=-\sum_{i}^{n}c_{i}\log\left(\frac{\text{exp}\left(z_{i}\right)}{\sum_{j}\text{exp}\left(z_{j}\right)}\right)$$

(5) $$L_{k}=\alpha L_{soft}+\left(1-\alpha \right)L_{hard}$$

The obtained final loss is in the form of backpropagation for the MSC-MobileViT update parameter.

### 4.4 Experimental equipment

The experiment uses a standardized computing platform for model development and validation, with the following hardware configuration: Intel Core i9-10980XE central processor (base frequency 3.0GHz), 64GB DDR4 memory, and NVIDIA GeForce RTX 3090 graphic processor (24GB GDDR6X video memory). The software environment is Windows 11 64-bit Professional operating system, the development tool is PyCharm 2021, the programming language is Python 3.9, and the deep learning framework is PyTorch 2.3.0 (CUDA acceleration support). All experiments were executed in a standalone GPU environment.

### 4.5 Model training and validation

#### 4.5.1 Training processes

To ensure that the experimental process is reproducible. The network training was set to 200 full training cycles (Epoch), and the batch size was kept at a constant ratio of 32 samples/batch. The optimizer chooses the stochastic gradient descent (SGD) method, the momentum factor is set to 0.9, and the weight decay is set to 5e-4. The learning rate of the feature extraction backbone network is set to 0.001, and its learning rate is set to 0.01, and the string annealing algorithm is used. Each Epoch automatically decays the learning rate to 1/1000 of the initial learning rate at the Meanwhile, this study adopts the migration learning technique in training the expert models by using each of the three expert models on the ImageNet dataset as a pre-training network and migrating its parameters as the starting point for training.

#### 4.5.2 Evaluation indicators

This study evaluates the model by F1-score, Accuracy, Precision, and Recall. Accuracy represents the probability that the predicted value is the same as the label value. Precision represents the probability that the label value is positive simultaneously in all samples with a positive predicted value. Recall represents the probability that the sample with a positive label value is predicted to be positive. The F1-score is the harmonic mean of accuracy and recall, and the evaluation performance is better when the data sample is unbalanced. The calculation formulas of the four evaluation indexes are Equations 6–9, where TP is the positive sample with a positive predictive value, FP is the negative sample with a positive predictive value, and FN is the positive sample with a negative predictive value.


(6) $$\text{Accuracy}=\frac{\sum_{i=1}^{n}TP_{i}/I_{i}}{n}$$

(7) $$Precision=\frac{TP}{TP+FP}$$

(8) $$Recall=\frac{TP}{TP+FN}$$

(9) $$F1-score=\frac{1}{\frac{1}{Precision}+\frac{1}{Recall}}=\frac{2\;\times \;Precision\;\times \;Recall}{Precision+Recall}$$

## 5 Results and discussion

### 5.1 Demonstration of the overall recognition effect of the model

The multi-expert grouping enforcing strategy coupled with the lightweight greenhouse tomato cultivation cycles long-tail identification method proposed in this study demonstrates significant performance advantages in the experiments. By deeply analyzing the experimental results demonstrated in **Table 3**, we can gain insight into the effectiveness of the method and its potential mechanism of action. First, all three expert models (Expert-head, Expert-balance, and Expert-tail) exhibit excellent performance on their respective test sets, confirming the grouping strategy’s effectiveness. By dividing the dataset by the number of samples, each expert model can focus on learning specific distributional features, thus achieving high accuracy on the subset for which each is responsible. Second, the Expert-ensemble model maintains a high level of performance, especially in terms of recall, although its overall performance is slightly lower than that of a single expert model. This phenomenon may stem from the fusion and trade-off of different expert knowledge in the ensemble process. Most striking is the performance of the MSC-MobileViT-distillation model. It not only outperforms the baseline model MSC-MobileViT in all metrics but also equals Expert-ensemble in accuracy and even achieves further improvement in the other three metrics. The above results highlight the potential of knowledge distillation techniques in model optimization. Knowledge distillation allows small models to learn from more complex models, which conveys critical information and serves as regularization to some extent.

**Table 3:** Experimental results for each expert model and MSC-MobileViT.

Classes in the test set	Model	Accuracy	Precision	Recall	F1-score
Idle Period, Mid Production, Others	Expert-head	98.09%	98.14%	98.11%	98.13%
Late Production, Seedling Pulling, Planting, Others	Expert-balance	94.48%	90.26%	94.28%	92.09%
Substrate Placement, Hole Opening, Substrate Soaking, Early Production, Others	Expert-tail	95.99%	75.17%	90.29%	80.53%
All Classes	Expert-ensemble	93.58%	86.69%	92.55%	88.64%
All Classes	MSC-MobileViT	92.68%	86.29%	85.01%	85.19%
All Classes	MSC-MobileViT- distillation	**95.99%**	**91.03%**	**93.57%**	**92.02%**

Bold values indicate the best performance for each metric.

The significant improvement in precision, recall, and F1 score of the MSC-MobileViT-distillation model reflects its strength in balancing the ability to recognize different categories. This may be attributed to the multi-scale knowledge inputs of the multi-expert model, allowing the student model to understand the data distribution more comprehensively. It is worth noting that MSC-MobileViT-distillation significantly improves the recall rate (by 8.56% compared to the baseline model) while maintaining high accuracy. This indicates that the model accurately recognizes common categories and effectively captures features of rare categories. This balance is essential for real-world application scenarios, especially in tasks such as cultivation cycles identification, which is sensitive to false omissions.

### 5.2 Effectiveness of multi-expert strategies

The multi-expert grouping enforcing strategy proposed in this study demonstrates significant advantages in the long-tail recognition task of greenhouse tomato cultivation cycles. By profoundly analyzing each expert model’s contribution and confusion matrix, we can understand the effectiveness of the strategy and its mechanism of action more comprehensively.

#### 5.2.1 Analysis of the contribution of each expert model

The effectiveness of the multi-expert joint guidance strategy can be analyzed by comparing the accuracies of the different models in the three category groups in **Table 4**. Significantly, the MSC-MobileViT differs in the recognition accuracy of the head and tail categories without expert model guidance, with a difference of 16.05 percentage points. In contrast, the Expert-ensemble model had a maximum difference of only 1.64 percentage points, suggesting that the grouping mechanism of the multi-expert model effectively balances attention to all categories, not just the head category. Further, by comparing the expert-ensemble model with the grouped form of the expert model, we can observe an improvement in both the BALANCE and the TAIL categories. At the same time, there is a decrease of 5.02 percentage points in the HEAD category compared to the Expert-head model, which suggests that the strategy effectively directs the model to pay more attention to the non-HEAD category, thus achieving a more balanced performance. Ultimately, the accuracy of the Expert-ensemble distillation-trained MSC-MobileViT exceeds the baseline level on all groups, especially the tail category, improves by 14.6 percentage points, and the maximum gap between different groups is only 3.49 percentage points.

**Table 4:** Accuracies of the different models in the three classes.

Model	Accuracy of head classes	Accuracy of balance classes	Accuracy of tail classes
Expert-head	98.33%		
Expert-balance		94.01%	
Expert-tail			91.46%
Expert-ensemble	93.31%	94.32%	92.68%
MSC-MobileViT	95.32%	91.17%	79.27%
MSC-MobileViT-distillation	**97.32%**	**94.32%**	**93.83%**

Bold values indicate the best performance for each metric.

First, from the analysis of the contribution of each expert model in **Table 4**, we observe a striking phenomenon: the recognition accuracy gap between the head category and the tail category of MSC-MobileViT without specialist guidance is as high as 16.05 percentage points. The colossal difference highlights the challenge of long-tailed distributed datasets for traditional model training. This phenomenon is consistent with the findings of Zhang et al. (2023c), who pointed out that on long-tailed distribution datasets, the model tends to favor the head category with a large sample size, significantly decreasing the ability to recognize the tail category. In contrast, the maximum accuracy difference of the Expert-ensemble model between different sets of categories is only 1.64 percentage points, and the above results fully demonstrate the effectiveness of the multi-expert grouping strategy in balancing the recognition ability of different categories. This significant improvement can be attributed to the grouping mechanism allowing each expert model to focus on learning specific distributional features, thus achieving a balanced focus on all categories overall.

Further comparing the performance of the expert ensemble with that of individual expert models, we find an improvement in the BALANCE category and the TAIL category and a decrease of 5.02 percentage points in the HEAD category compared to the Expert-head model. This trade-off phenomenon reflects the strategy’s success in directing the model to pay more attention to non-head categories, achieving a more balanced performance. The above results echo the study of Wang et al. (2022), who suggested that appropriately reducing the focus on head categories can significantly improve overall performance when dealing with unbalanced data.

Most notably, the accuracy of the Expert-ensemble distillation-trained MSC-MobileViT-distillation model exceeds the baseline level on all category groups, especially the tail category, improves by 14.6 percentage points, and the maximum gap between different groups is only 3.49 percentage points. The above results fully demonstrate the effectiveness of knowledge distillation in transferring expert model ensemble knowledge. This significant performance improvement may stem from the fact that during the distillation process, the student model not only learns the complex labels but also captures the rich information contained in the soft output of the teacher model, which is consistent with the theory of knowledge distillation proposed by Hinton (2015).

#### 5.2.2 Analysis of confusion matrices

**Figure 7:**
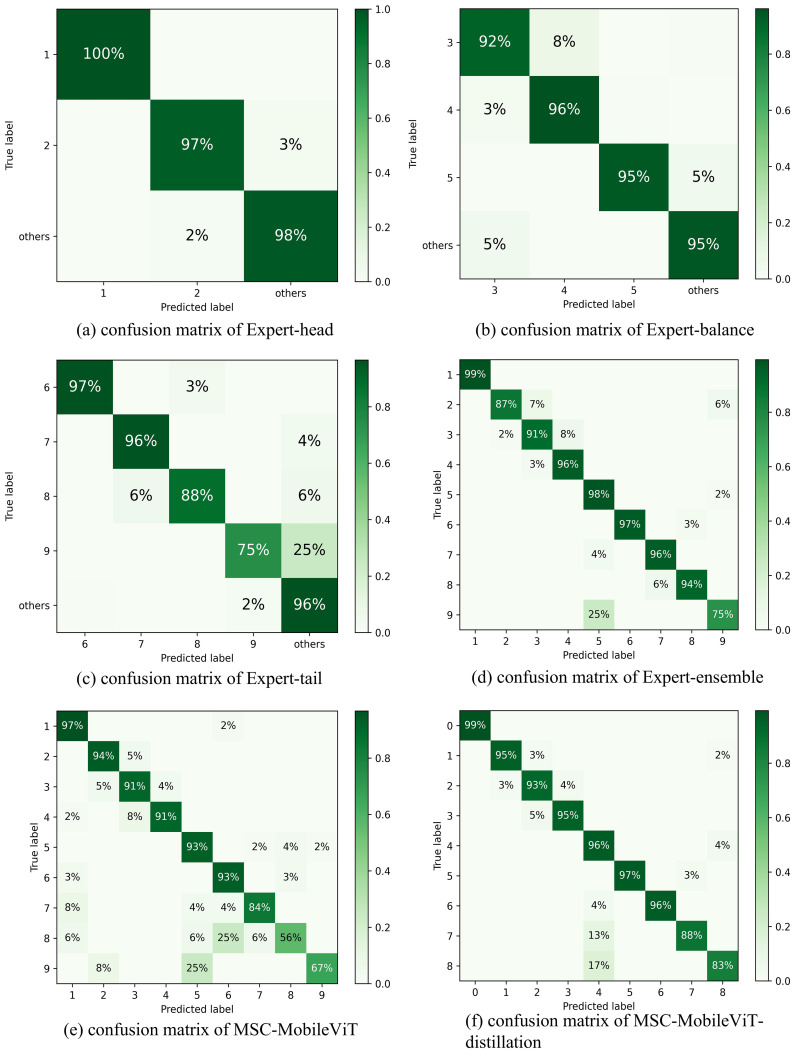
The confusion matrices of **(a)** Expert-head, **(b)** Expert-balance, **(c)** Expert-tail, **(d)** Expert-ensemble, **(e)** MSC-MobileViT, and **(f)** MSC-MobileViT-distillation on the testing dataset. Note: 1 Idle Period; 2 Mid Production; 3 Late Production; 4 Seedling Pulling; 5 Planting; 6 Substrate Placement; 7 Hole Opening; 8 Substrate Soaking; 9 Early Production.

The analysis of the confusion matrix further corroborates the effectiveness of the multi-expert strategy, as shown in **Figure 7**. The low accuracy of MSC-MobileViT on the tail categories of Substrate Soaking and Early Production (56% and 67%, respectively) highlights the negative impact of long-tailed distributions on the performance of the model, i.e., on unbalanced datasets that minority classes are often misclassified as majority classes. In contrast, MSC-MobileViT-distillation trained through the joint guidance of multi-expert models not only outperforms Expert-ensemble and MSC-MobileViT in terms of overall accuracy but also improves the accuracy of the Substrate Soaking and Early Production classes by up to 88% each. Accuracy was enhanced to 88%, 83%, 32%, and 16%, respectively. This significant improvement may be attributed to the success of the multi-expert strategy in effectively transferring the expertise of different expert models in their respective domains to the student model. However, it is worth noting that although MSC-MobileViT-distillation achieved significant improvements in all categories, there is still room for improvement in some categories. For example, although substantially improved, the Substrate Soaking category’s accuracy is still lower than other categories. This may imply that the feature representation of some extremely unbalanced categories may not be fully captured during the knowledge distillation process. Future research could explore optimizing the distillation process further to deliver knowledge of rare categories more efficiently.

In summary, the multi-expert grouping enforcing strategy proposed in this study mitigates the challenges posed by long-tailed distribution datasets by effectively balancing the recognition capabilities of different categories. The approach theoretically bridges the advantages of expert systems and knowledge distillation and demonstrates its applicability in practice in complex scenarios. This provides a new way of thinking to solve the long-tailed recognition problem prevalent in industrial and agricultural fields and lays the foundation for exploring similar strategies in a broader range of application scenarios in the future.

### 5.3 MSC-MobileViT benchmark performance analysis

This study provides insights into the performance advantages of the MSC-MobileViT model in the greenhouse tomato working long-tail identification task through ablation experiments and Grad-CAM visualization analysis.

#### 5.3.1 Ablation analysis

This paper provides an in-depth analysis of the self-attention mechanism and the advantages of multi-scale modules for saliency region feature extraction. We found significant differences by comparing the performance of three models, MobileNetV3, MobileViT, and MSC-MobileViT. MobileNetV3, as a convolutional network, contains the classical SE attention mechanism module, which makes it capable of efficiently processing spatial information. However, MobileViT introduces a transformer architecture, and the above innovation enables it to capture long-range dependencies, which improves the model’s ability to understand global information. On this basis, MSC-MobileViT further integrates a multi-scale module, and the above improvement enables the network to extract features at different scales, allowing the model to focus on both details and overall structure, which leads to a more comprehensive understanding of the image content and enhances the model’s expressive power. **Table 5** shows that MobileViT outperforms MobileNetV3 in terms of performance, while MSC-MobileViT achieves further improvements based on MobileViT. These findings confirm the importance and effectiveness of self-attention mechanisms and multi-scale modules in enhancing network performance.

**Table 5:** Analysing the advantages of MSC-MobileViT.

Model	Accuracy	Precision	Recall	f1-score
MobileNetV3	91.47%	81.73%	78.58%	78.34%
MobileViT	92.38%	85.39%	79.86%	77.54%
MSC-MobileViT	**92.68%**	**86.29%**	**85.01%**	**85.19%**

Bold values indicate the best performance for each metric.

First, the results of the ablation experiments demonstrate the gradual performance improvement process from MobileNetV3 to MobileViT to MSC-MobileViT. MobileNetV3, as the benchmark model, integrates the SE attention mechanism, but it has an accuracy of 91.47% and a relatively limited performance on long-tailed distribution datasets.

MobileViT, by introducing the Transformer architecture, improves the accuracy to 92.38%, and the precision and recall rates are also significantly improved. This performance improvement may stem from the ability of the Transformer architecture to capture long-range dependencies, which echoes the “ Attention Is All You Need” theory proposed by Vaswani et al. (2017). In a complex task like greenhouse tomato cultivation cycles identification, the importance of global contextual information cannot be overstated, and Transformer’s self-attention mechanism can capture this information effectively.

MSC-MobileViT further improves the model performance based on MobileViT by integrating the multi-scale module, especially regarding recall and F1 score. The accuracy reached 92.68%, recall improved by 5.15 percentage points, and F1 score improved by 7.65 percentage points. This overall performance improvement may be attributed to the ability of the multiscale module to extract features at different scales, thus focusing on both local details and the global structure of the image.

#### 5.3.2 Grad-CAM visualization and analysis

**Figure 8:**
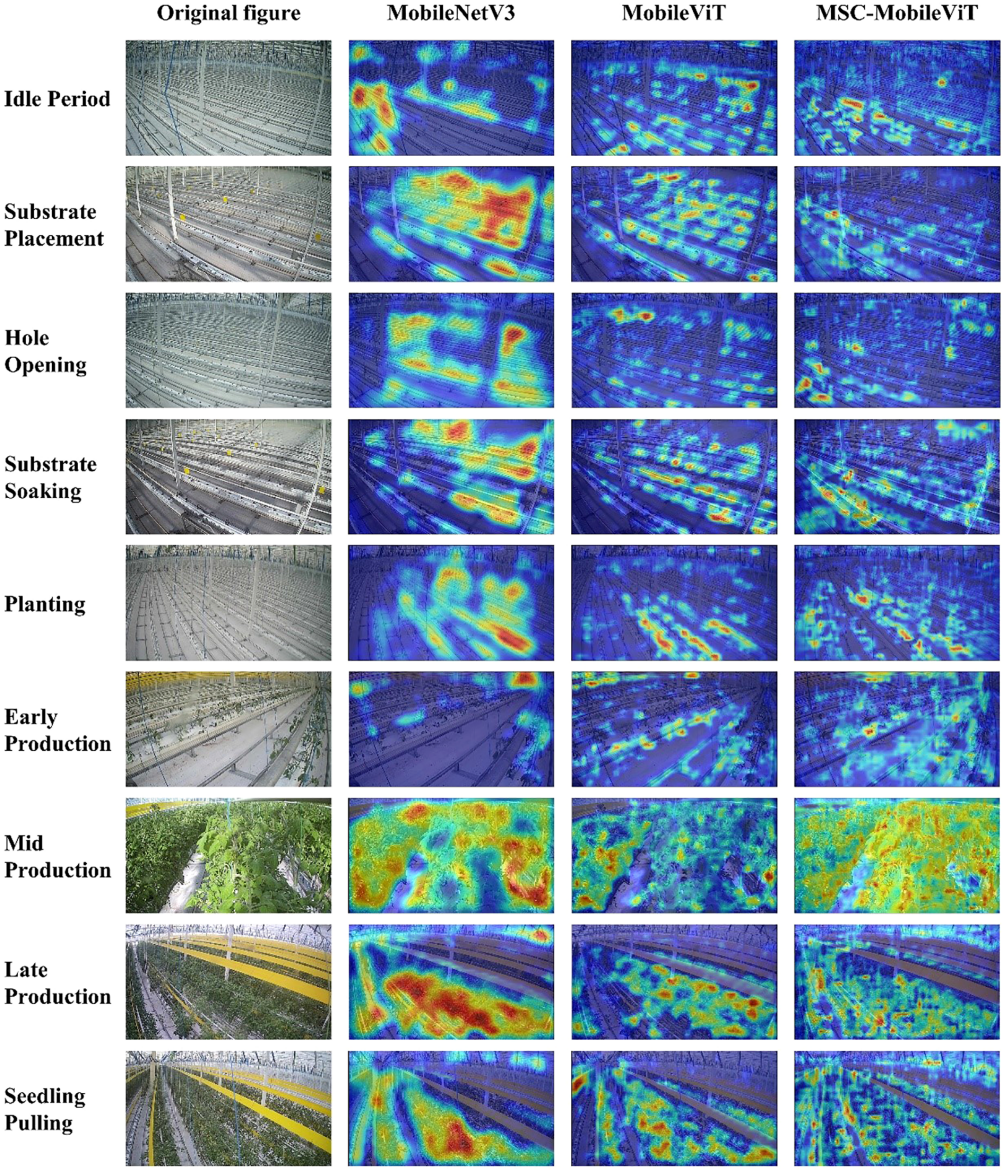
Grad-CAM visualization.

The Grad-CAM visualization analysis corroborates the above findings while providing more profound insights. The heatmap generated by MobileNetV3 shows that the model tends to focus on more significant contiguous regions **Figure 8**, which reflects the advantage of convolutional networks in capturing local features. However, this focus pattern may result in some critical long-range dependencies being overlooked, which is consistent with the finding of Jiang et al. (2024) that traditional CNNs may over-focus on certain discriminative regions while ignoring other important information. In contrast, MobileViT’s heatmap presents a more decentralized and fine-grained distribution of concerns, suggesting that the Transformer architecture can better capture global contextual information. This feature is essential when dealing with complex scenarios, such as identifying tomato plants at different cultivation cycles.

The heatmap of MSC-MobileViT shows the most detailed and diverse feature focus patterns. In recognition of complex categories such as “Mid Production,” MSC-MobileViT can focus on the plant’s overall structure and the local details, such as leaf morphology, fruit status, etc. This multi-scale feature extraction capability is critical when recognizing complex scenes, such as tomato plants at different stages of growth. This multi-scale feature extraction capability not only improves the recognition accuracy of the model but also enhances its sensitivity to slight differences in various cultivation cycles. The above findings echo the Multiscale Visual Transformer (MViT) proposed by Fan et al. (2021), who emphasized the importance of multiscale feature learning for improving the performance of visual tasks.

### 5.4 Stratified cross-validation

To ensure a more robust evaluation of our model’s generalization stability and reduce the bias that can stem from a single data split, we employed five-fold stratified cross-validation in **Table 6**. We chose an 8:2 split between training and validation sets without applying data augmentation. While the original setup used a 1:1 split and included augmentation, this difference might introduce some variability in results. However, the overall performance trend remains consistent.

**Table 6:** The stratified cross-validation.

Folds	Accuracy		Precision		Recall		f1-score	
	baseline	ours	baseline	ours	baseline	ours	baseline	ours
K1	94.29%	96.03%	88.69%	93.81%	85.84%	91.21%	86.83%	92.34%
K2	94.28%	95.88%	89.91%	91.45%	89.05%	91.70%	89.11%	90.49%
K3	94.76%	95.71%	87.17%	89.46%	82.50%	93.70%	83.90%	91%
K4	95.23%	97.24%	89.60%	90.69%	87.97%	94.02%	89.12%	91.78%
K5	94.43%	95.44%	85.51%	90.63%	83.21%	92.10%	83.21%	91.06%
mean	94.60%	96.06%	88.18%	91.21%	85.71%	92.55%	86.43%	91.33%

Across all key metrics, our approach (Ours) consistently outperformed the baseline model. The average accuracy reached 96.06%, representing a 1.46 percentage point improvement over Baseline’s 94.60%. Fold K4 was particularly notable, where accuracy rose to 97.24%, 2.01 points above the baseline. This suggests that when more data is available, our model avoids overfitting and better captures the underlying distribution. Even in Fold K1, which included fewer rare categories, our model still achieved an impressive 96.03% accuracy, outperforming the baseline by 1.74 points and demonstrating strong adaptability.

Recall, which is especially important for identifying long-tail categories, also improved substantially. The average recall for our model was 92.55%, a 6.84 point increase over the baseline. In Fold K3, recall reached 93.70%, outperforming the baseline by over 11 points. In Fold K4, the difference was 6.05 points. These gains highlight the effectiveness of the multi-expert grouping strategy, which helps the model learn underrepresented features more effectively—even without augmentation. Stages such as Substrate Soaking and Early Production, which have fewer samples, were better captured under this approach.

Precision and F1-score saw similar improvements. Our model averaged 91.21% for precision and 91.33% for F1-score, outperforming the baseline by 3.03 and 4.90 points respectively. In Fold K3, for instance, precision was 89.46% compared to the baseline’s 87.17%, indicating fewer false positives. Fold K4 showed an F1-score of 91.78%, outpacing the baseline by 2.66 points and demonstrating a well-balanced trade-off between accuracy and recall.

Model stability is especially critical in real-world deployment scenarios. Our results showed smaller variation across folds, with a standard deviation of 0.62% for accuracy and 1.11% for recall. In contrast, the baseline showed higher variability—0.36% and 2.56%, respectively—suggesting that our approach yields more consistent performance across diverse subsets such as seasonal or greenhouse-specific data.

### 5.5 Comparison with existing methods

#### 5.5.1 Comparison with the main network of other classifications

This study compares the performance of multiple state-of-the-art models in a long-tail recognition task for greenhouse tomato cultivation cycles, and the experimental results are shown in **Table 7**. Overall, the accuracy of all models ranges from 83.25% to 95.99%, showing a significant performance variation. Our proposed model performs best in critical metrics such as accuracy, precision, recall, and F1 score while maintaining deficient parameters, demonstrating an excellent performance-efficiency balance.

**Table 7:** Comparison experiment with the main network of other classifications.

Model	Accuracy	Precision	Recall	f1-score	Parameters	FLOPs
ViT16	87.86%	81.79%	75.74%	76.96%	50.07M	11.29G
ResNet50	83.25%	52.39%	51.36%	51.52%	25.56M	4.13G
MobilenetV3	91.47%	81.73%	78.58%	78.34%	4.21M	**0.23G**
MobileOneS0	86.96%	67.35%	65.17%	64.94%	4.28M	1.12G
MobileOneS1	86.36%	72.68%	64.16%	62.74%	3.56M	0.89G
MobileVit-s	93.88%	88.05%	86.55%	87.12%	4.94M	1.46G
MobileVit-xs	89.77%	70.63%	71.23%	70.22%	1.94M	0.74G
MobileVit-xxs	92.38%	85.39%	79.86%	77.54%	**0.95M**	0.27G
Ours	**95.99%**	**91.03%**	**93.57%**	**92.02%**	**0.95M**	0.29G

Bold values indicate the best performance for each metric.

From the accuracy perspective, our model tops the list with 95.99%, outperforming all compared models. The MobileVit-s model follows with 93.88% accuracy, while the ResNet50 model performs poorly with only 83.25%. Notably, despite having the most significant number of parameters (50.07M), the ViT16 model fails to match the accuracy (87.86%) of the lighter models with far fewer parameters, highlighting the critical impact of model design on performance.

In terms of accuracy, our model also performs well with 91.03%. The MobileViT-s model comes in second place with 88.05% accuracy, while the ResNet50 model is again at the bottom of the list with 52.39%. The above results reflect traditional convolutional neural networks’ challenges when dealing with long-tailed distributed datasets.

On the recall metric, our model is significantly ahead of the others with an excellent result of 93.57%. The MobileVit-s model comes in second place with 86.55%, while the ResNet50 model performs poorly with a recall of only 51.36%. The above results highlight the superior ability of our proposed model to recognize various types of cultivation cycles, especially rare categories.

The F1 score, as a reconciled average of precision and recall, reflects the model’s overall performance more comprehensively. Our model leads the pack with 92.02% on the above metrics, and MobileVit-s comes in second with 87.12%. Interestingly, although MobilenetV3 performs well in accuracy (91.47%), its F1 score (78.34%) is relatively low, which may hint at some model limitations in dealing with unbalanced datasets.

Regarding the number of parameters, our method has only 0.95M parameters, which is tied with MobileVit-xxs for the lowest but significantly outperforms the latter. In contrast, ViT16 and ResNet50 have 50.07M and 25.56M parameters, respectively, yet fail to dominate in performance, highlighting our model’s outstanding advantage in balancing efficiency and performance.

The model proposed in this study performs well in the greenhouse tomato cultivation cycles long-tail recognition task, comprehensively outperforming existing state-of-the-art models in all performance metrics and achieving a meager parametric count.

First, the significant advantages of our model in terms of accuracy (95.99%) and recall (93.57%), especially compared to MobileVit-xxs with a similar number of participants (92.38% accuracy and 79.86% recall), highlight the effectiveness of our proposed multi-expert grouping enforcing strategy. This performance improvement may stem from the strategy effectively mitigating the category imbalance problem caused by the long-tailed distribution.

Second, our model keeps the number of parameters at 0.95M while maintaining high performance, which is significant in lightweight model design. In contrast, ViT16 has a 50.07M parameter count, but its accuracy (87.86%) and F1 score (76.96%) are significantly lower than our model. Our approach may have achieved more efficient parameter utilization by combining multi-scale feature extraction and knowledge distillation, thus capturing richer feature representations within a limited parameter space.

Notably, our model’s F1 score (92.02%) advantage over other metrics is more prominent. The above results imply that the model improves the overall accuracy and achieves a better balance in all categories when dealing with long-tailed distribution data. This balance is crucial for practical applications, especially in tasks such as cultivation cycles identification, which is sensitive to misidentification.

Another point of concern is the poor performance of the ResNet50 model in this task (83.25% accuracy, 51.52% F1 score). The above results may reflect the limitations of traditional convolutional neural networks when dealing with long-tailed distribution data and complex scene recognition tasks. In contrast, models based on the Transformer architecture (e.g., the MobileVit family and our model) generally perform better, which may be attributed to the advantage of the self-attention mechanism in capturing long-range dependencies.

In addition, we expanded the evaluation to include parameter count and computational cost, showing that MobileViT-S achieved 93.88% accuracy and an 87.12% F1 score with 4.94 M parameters and 1.46 G FLOPs while our model reached 95.99% accuracy and a 92.02% F1 score using 0.95 M parameters and 0.29 G FLOPs. Plotting accuracy against FLOPs positioned our approach on the Pareto frontier, evidencing an ideal balance between resource efficiency and predictive performance. Future work might explore dynamic early-exit mechanisms, hardware-aware neural architecture search, mixed-precision quantization, and knowledge-distillation techniques to push sub-1 M-parameter models beyond 0.3 G FLOPs without sacrificing accuracy.

#### 5.5.2 Comparison with SOTA long-tailed recognition methods

To further assess the advantages of our approach in long-tail recognition, we conducted a systematic comparison against the Bilateral Branch Network (BBN) (Zhou et al., 2020), the Re-mixing Strategy(ReMix) (Chou et al., 2020), Balanced Margin Softening (BMS) (Ren et al., 2020), and the Curvature-Balanced Feature Manifold Learning Method(CR) (Ma et al., 2023) in **Table 8** Comparison experiment with SOTA long-tailed recognition methods. The results show that our method delivers the highest accuracy on tail categories. For the extremely rare Substrate Soaking stage, we achieve an identification rate of 87.50%, matching BMS. In the Early Production stage, we set a new record with 83.33% accuracy—an 8.33-point improvement over the next best method. Hole Opening and Substrate Placement attain 96.00% and 96.55%, respectively, confirming that the group expert strategy represents a substantial breakthrough in modeling rare samples.

**Table 8:** Comparison experiment with SOTA long-tailed recognition methods.

Groups	Classes	BBN	ReMix	BMS	CR	Ours
Head Classes	Idle Period	92.76%	98.68%	97.70%	98.36%	**99.34%**
	Mid Production	93.20%	**96.26%**	94.56%	**96.26%**	95.24%
Balance Classes	Late Production	87.02%	84.73%	88.55%	87.02%	**93.13%**
	Seedling Pullin	88.46%	91.54%	94.62%	**95.38%**	94.62%
	Planting	78.57%	89.29%	92.86%	94.64%	**96.43%**
Tail Classes	Substrate Placement	93.10%	**96.55%**	89.66%	**96.55%**	**96.55%**
	Hole Opening	80%	92%	88%	88%	**96%**
	Substrate Soaking	37.50%	75%	**87.50%**	62.50%	**87.50%**
	Early Production	75%	75%	75%	50%	**83.33%**
All		89.37%	93.78%	93.98%	94.18%	**95.99%**

Bold values indicate the best performance for each metric.

Overall, our method secures a leading position with an average accuracy of 95.99%, outperforming the second-place CR (94.18%) by 1.81 points. Importantly, this advantage extends beyond tail categories: in the Planting stage (balanced categories), we reach 96.43%—far exceeding existing state-of-the-art methods and demonstrating that knowledge distillation technology effectively integrates expert knowledge across groups. For head categories, the Idle Period stage achieves an exceptional 99.34% accuracy, illustrating a new paradigm of head-tail collaborative optimization.

### 5.6 Limitations and future work

#### 5.6.1 Research limitations

Despite the significant results achieved in this study in the task of long-tail identification of greenhouse tomato cultivation cycles, there are still some limitations of concern. First, the limitations of the dataset may affect the model’s generalization ability. Although covering multiple cultivation cycles categories, the dataset used in this study may not fully reflect all possible real-world production scenarios, especially some extreme or rare cases. Second, although our model performs well on the current dataset, its ability to generalize to other environments or conditions has yet to be verified. Finally, although our model achieves few parameters, it may face computational resource constraints in real-world deployments, especially on resource-constrained edge devices.

#### 5.6.2 Future Research Directions

Based on these limitations, we propose the following promising directions for future research:

1. Enhanced data diversity and adaptive learning: future research could focus on constructing a more representative and diverse dataset of greenhouse tomato cultivation cycles, including different varieties, growth stages, and anomalies. At the same time, explore adaptive learning algorithms that enable the model to continuously learn from and adjust to new data, thus improving its ability to generalize in dynamic environments.
2. Optimize the multi-expert collaboration mechanism: conduct in-depth research on improving the multi-expert grouping enforcing strategy to handle the unbalanced long-tailed distribution better. Dynamic expert allocation mechanisms or meta-learning approaches can be explored to enable models to adjust expert combinations according to different data distribution characteristics automatically. Such approaches may provide new ideas for solving the problem of the generalizability of AI systems.
3. Cross-modal learning and knowledge fusion: consider extending this study to multimodal learning, combining multi-source information such as image data, environmental sensor data, and plant physiological indicators to construct a more comprehensive cultivation cycles recognition system. This will not only improve the accuracy and robustness of the recognition but may also reveal new plant growth patterns and provide deeper insights into innovative agricultural management.
4. Edge Intelligence and Federated Learning: To address the limitations of practical deployment environments, we study how to lighten the model further and, at the same time, explore federated learning techniques to achieve distributed model training and updating. This can fully use decentralized computational resources while protecting data privacy to realize large-scale, real-time work monitoring systems.

Through these research directions, we aim to promote the development of innovative agriculture technology further, extend the results of this study to a broader range of application scenarios, and ultimately realize a more efficient and sustainable agricultural production model.

## 6 Conclusion

This study proposes an innovative lightweight recognition model coupled with a multi-expert grouping enforcing strategy for the above challenging problem of long-tailed recognition of greenhouse tomato cultivation cycles. Through in-depth theoretical analysis and experimental validation, we draw the following core conclusions:

1. The multi-expert grouping enforcing strategy significantly improves the recognition performance of long-tailed distribution data: our approach successfully improves the recognition accuracies of tailed categories (e.g., Substrate Soaking and Early Production) from 56% and 67% in the baseline model to 88% and 83%, which fully demonstrates the effectiveness of the strategy in alleviating the category imbalance problem. The above findings provide a new paradigm for dealing with long-tailed distribution data, which is applicable to agriculture and may also be extended to other fields with category imbalance problems.
2. Knowledge distillation technique realizes the balance between high performance and low number of parameters. By effectively transferring the knowledge from multiple expert models into a lightweight student model with only 0.95M parameters, our method significantly reduces the computational complexity while maintaining high performance (95.99% accuracy and 92.02% F1 score). The above results provide a feasible solution for efficient AI deployment in resource-constrained environments and promote the development of edge intelligence technology.
3. The improved MSC-MobileViT model shows excellent feature extraction capability. By introducing a multi-scale convolutional module, our model can capture both local details and the global structure of an image and performs well in complex scene recognition tasks. The above improvements not only improve the recognition accuracy of the model but also enhance its ability to adapt to features at different scales, which provides new ideas for model design in the field of computer vision.

The findings of this study have important theoretical and practical implications for the fields of intelligent agriculture and computer vision. Theoretically, our study expands the long-tailed distribution data processing methodology and provides a new perspective for solving the category imbalance problem. At the practical level, our approach provides a powerful tool for intelligent management of greenhouse tomato production, which has the potential to improve production efficiency and resource utilization significantly.

## Data Availability

The datasets presented in this article are not readily available because Image datasets are required to be kept confidential. Requests to access the datasets should be directed to xuf@nercita.org.cn.
